# Gyrocardiography: A New Non-invasive Monitoring Method for the Assessment of Cardiac Mechanics and the Estimation of Hemodynamic Variables

**DOI:** 10.1038/s41598-017-07248-y

**Published:** 2017-07-28

**Authors:** Mojtaba Jafari Tadi, Eero Lehtonen, Antti Saraste, Jarno Tuominen, Juho Koskinen, Mika Teräs, Juhani Airaksinen, Mikko Pänkäälä, Tero Koivisto

**Affiliations:** 1 University of Turku, Faculty of Medicine, Turku, Finland; 2 University of Turku, Department of Future Technologies, Turku, Finland; 3 Turku University Hospital, Heart Center, Turku, Finland; 4 University of Turku, Institute of Biomedicine, Turku, Finland; 5 Turku University Hospital, Department of Medical physics, Turku, Finland

## Abstract

Gyrocardiography (GCG) is a new non-invasive technique for assessing heart motions by using a sensor of angular motion – gyroscope – attached to the skin of the chest. In this study, we conducted simultaneous recordings of electrocardiography (ECG), GCG, and echocardiography in a group of subjects consisting of nine healthy volunteer men. Annotation of underlying fiducial points in GCG is presented and compared to opening and closing points of heart valves measured by a pulse wave Doppler. Comparison between GCG and synchronized tissue Doppler imaging (TDI) data shows that the GCG signal is also capable of providing temporal information on the systolic and early diastolic peak velocities of the myocardium. Furthermore, time intervals from the ECG Q-wave to the maximum of the integrated GCG (angular displacement) signal and maximal myocardial strain curves obtained by 3D speckle tracking are correlated. We see GCG as a promising mechanical cardiac monitoring tool that enables quantification of beat-by-beat dynamics of systolic time intervals (STI) related to hemodynamic variables and myocardial contractility.

## Introduction

The heart is an intricate object which undergoes repeating changes in different dimensions and orientations^1^. The contraction of helically oriented muscle fibres act as an integrated force causing a coordinated wringing motion to the myocardium within each cardiac cycle^2^. Accordingly, the longitudinal retraction of the myocardium causes left ventricular (LV) base to move towards apex. Linear contribution of the muscle fibres contraction in the long axis of the heart is known as an indicator of ventricular systolic and diastolic mechanical function^3, 4^.

Monitoring of the myocardial mechanical activity requires sophisticated techniques. Over the past years, cardiac motion has been widely studied and quantitatively investigated using invasive and non-invasive techniques in both animals and humans. In 1975, Ingels *et al*.^5^ proposed an invasive method to evaluate LV performance in dogs based on multiple implanted radiopaque markers and biplane cine angiography analysis. Later, other non-invasive approaches based upon optical devices^6, 7^, tagged magnetic resonance imaging (tagged MRI)^8^, tissue Doppler imaging (TDI)^9^ and speckle tracking imaging^10^ were introduced in order to evaluate dynamics of cardiac motion and myocardial tissue function.

Ballistocardiography (BCG) — the recording of the reactionary forces of the body invented by Gordon in 1877 – and seismocardiography — the recording of chest wall vibrations invented by Bozhenko in 1961 — are non-invasive methods which have been used for cardiac mechanical monitoring^11–14^. In principle, BCG measures the whole body recoil or ballistic forces in response to the blood ejection from aorta into the vascular tree, while the SCG measures the positional vibrations of the chest wall in reaction to the myocardial motions and respiration^15, 16^. In a recent study, sophisticated *in vivo* experimental examinations and a complementary mathematical model revealed that BCG waves are formed due to blood pressure gradients in the ascending and descending aorta^17^. SCG and BCG, which are typically based on using accelerometers and force sensors, can be used for unobtrusive long term monitoring of LV to estimate hemodynamic variables, cardiac abnormalities, and breathing disorders via low-cost wearable or portable devices^18–26^. Recent studies have also briefly described the feasibility of heart monitoring using built in accelerometer and gyroscope sensors in Google glasses, wrist worn devices, smart phones, and chest worn patches^27–30^. Additionally, the body kinetic energy analysis, also known as the multi-dimensional kineticardiography (MKCG), has been recently introduced in ref. 31. MKCG is based on placing a tri-axial accelerometer and a two-axial gyroscope on the center of mass of the body, and on thereby measuring the rotational and translation kinetic energies and powers of the body. This method has been shown to be useful in evaluating kinetic energy transferred from the heart in patients suffering valvulopathy and heart failure^31^. Marcelli *et al*.^32, 33^, Hyler *et al*.^34^, and Grymyr *et al*.^35^, on the other hand, reported invasive techniques based upon implantable gyroscope and accelerometer sensors in order to monitor left ventricular function and assess cardiac rotation in animals. These studies report promising results which may yield to a prospective strategy suitable for implantable devices for the continuous monitoring of cardiac function. Other studies have also showed that by using gyroscope one can improve the automated interpretation of SCG signals in order to estimate heart rate variability, cardiac time intervals and annotation of waveforms^36–38^.

Complementary to the above methods, we introduce in this paper a novel non-invasive approach for the measurement of cardiac and respiration signals we call gyrocardiography (GCG). This technique is solely based on measuring the precordial microvibrations using a microelectromechanical (MEMS) gyroscope sensor attached to the skin anterior to the sternum. GCG can be used to estimate beat-to-beat hemodynamic variables such as heart rate and the pre-ejection period, and to investigate the mechanical activity of the heart. The gyroscope measures its own angular velocity, and in this paper we present how some of the maxima or minima of these velocities correspond to physiological events such as the moments of LV valvular openings and closings. A benefit of the proposed method is that a gyroscope is a relatively cheap sensor, and is available for example in most wearable devices (e.g. wrist worn devices, smart phones, etc). However, the engagement mechanism and the transfer function from the motion of the heart to the motion of the chest are still unclear, and should be investigated more thoroughly in the future.

This paper is based on our previous publication^39^ in which we presented primary investigations on the GCG signal and its correspondence to the mechanical activity of the heart. In^40^ we also briefly demonstrated the feasibility of recovering respiration signals by using a gyroscope sensor for nuclear medicine imaging applications. In this work, we focus on exploring the correspondence between a reference echocardiography/cardiac ultrasound (US) and tri-axial GCG measurements in order to show that GCG is capable for estimating certain myocardial motions and hemodynamic variables. We present an annotation of major fiducial points in the GCG signal based on the timings of cardiophysiological events measured by a pulse wave Doppler. We also compare GCG signals to tissue velocity (TV) and strain curves obtained by TDI and speckle tracking analyses, and show that the timing of the maximal strain is correlated with certain waveform in the GCG signal. Furthermore, we present complementary information on automated heartbeat detection and annotation of the GCG signal that allows for estimating beat-to-beat cardiac time intervals (see Supplementary material). The accuracies of the annotations and other measurements are determined by statistical analysis.

## Methods

### Experimental Set-up and Protocols

In the following we describe our experimental set-up and measuring protocol employed for data collection and analysis.

#### Study subjects

**Figure 1:**
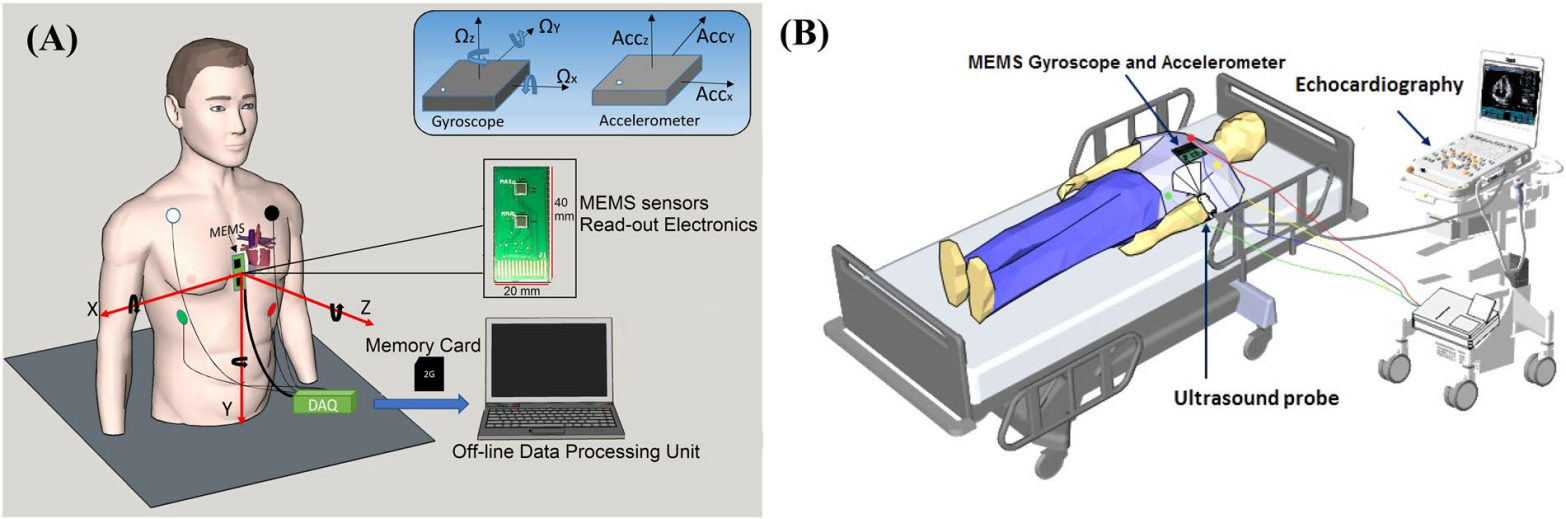
Simultaneous data acquisition from echocardiography, ECG, and MEMS sensors. General schematics of MEMS motion processing system (**A**) and echocardiography set up including MEMS sensors and ECG (**B**).

Experimental verification of the proposed approach was performed on the data acquired from 9 healthy volunteer subjects with their informed consent. All experiments were supervised in a controlled research environment and were performed in accordance with the Helsinki Declaration. We considered healthy male subjects who had no prior history of cardiovascular disease. The study subjects were asked to lie in the supine position with the upper body slightly tilted in order to facilitate echocardiography. Data was acquired simultaneously from the electrocardiography (ECG), the inertial measurement unit, and from the echocardiography in a time frame of approximately 10–15 minutes. Figure 1 shows the general measurement set-up utilized in this study. An expert echocardiographer who is also a cardiologist performed all the ultrasound examinations. Afterwards, visual data inspection was performed by three independent observers including one cardiologist. The demographic information of the study subjects as well as echocardiography characteristics are provided in Table 1.

**Table 1:** Demographic information and descriptive analysis of the echocardiography characteristics.

Demographic information	Min	Max	Mean	±SD	Echocardiography characteristics	Min	Max	Mean	±SD
Age (years)	23	46	31	8.3	LVEDV (ml)	67	154	118	25
Height (cm)	172	186	179	4.7	LVESV (ml)	31	58	47	8
Weight (kg)	70	85	76	5.8	EF (%)	54	63	59	3
BMI (kg/m^2^)	21.6	26.12	23.8	1.5	SV (ml)	59	96	73	13
Blood Pressure (mmHg)	119/70	165/87	130/77	14/5	CO (l/min)	3.1	5.1	4	0.75

*LVEDV = left ventricular end diastolic volume; LVESV = left ventricular end systolic volume; EF = ejection fraction; SV = stroke volume; CO = cardiac output; BP = blood pressure.

#### Data Acquisition

We used a custom-made miniaturized customized joint accelerometer-gyroscope system – inertial measurement unit (IMU) – in order to measure externally heart mechanical motions. Toward this end, a (3 mm × 3 mm × 1 mm) triple-axis, low-power, capacitive digital accelerometer (Freescale Semiconductor, MMA8451Q, Austin, TX, USA) and an (3 mm × 3 mm × 0.9 mm) ultra-accurate, low power, low noise, 3-axis angular rate sensor (Maxim Integrated, MAX21000, San Jose, CA, USA) were employed for recovering chest cardiac and respiratory signals. The MEMS sensors were attached to the skin of the chest anterior to the body of sternum using double-sided tape without hair removal in the chest area. The measured acceleration and angular velocity range of the accelerometer and gyroscope were set to ±2*g* and ±250 dps, respectively. The accelerometer has an RMS noise of 99 $\mu g/\sqrt{Hz}$ and is tuned to have an output bandwidth of 400 Hz, while the gyro low noise density was 9 $mdps/\sqrt{Hz}$ and the output bandwidth was 400 Hz. Additionally, a reference standard two lead front-end electrocardiogram (ADS1293 from Texas Instruments) was added to this prototype. All measurements were collected using FRDM-KL25Z (from Semiconductor) board, and stored on a memory card, and later were parsed and processed using a custom-made software. All GCG, SCG, and ECG data were recorded simultaneously with a sampling frequency (*Fs*) of 800 Hz. A 4^*th*^ order Butterworth IIR filter with pass bands 1–20 Hz and 4–45 Hz, respectively, were applied on the gyroscope and accelerometer derived signals, allowing the removal of white noise and signals offset. ECG signals were also de-noised by a fast Fourier transform (FFT) filter and with the frequency bands of 0.5–45 Hz as described in ref. 41. In addition to the above sensors, in a pre-study examination we considered three other gyroscope sensors, namely Murata SCC1300d02, Bosch BMI 160, and the SONY Xperia Z3 compact smartphone with a built in IMU in order to evaluate the reproducibility of the GCG waveforms. The sampling rate for the Murata sensor was set to 2000 Hz, while the other two sensors had the sampling rate of 200 Hz. Measurements with these sensors were performed for visual evaluation of inter- and intra-subject variability in the GCG waveform. Figure 1A shows the general measurement set up showing the location of tri-axial MEMS gyroscope and accelerometer for heart monitoring. Chest-attached (sternal) tri-axial MEMS accelerometer and gyroscope sensors (40 mm × 20 mm) and ECG body electrodes (white, green, black, and red) are wired to the data acquisition (DAQ) system. Red and black colour arrows show orientation of sensitivity and the polarity of the tri-axial MEMS sensors.

#### Echocardiography

The echocardiography examination was conducted by a Vivid E95 scanner with a 1.4–4.6 MHz transducer (GE Healthcare, Finland). A complete echocardiographic study was performed using standard apical views for 3 to 6 cardiac cycles. EchoPAC post-processing software (Version 113, GE Healthcare, Finland) was employed for off-line echocardiographic analysis of TDI and 3D speckle tracking strain. Conventional echocardiography, electrocardiography, and 3-axis GCG and 3-axis SCG were performed concurrently. For measurement of cardiac time intervals, mitral valve and aortic valve flow velocities were recorded using pulsed-wave (PW) Doppler. For measurement of myocardial velocities, apical 4 chamber TDI images were obtained with an average rate of 106/sec fps. We performed speckle tracking which is an automated functional imaging technique for multidimensional deformation or strain analysis. 3D volume covering the whole left ventricle myocardium was obtained from an apical view averaging 6 cardiac cycles with an average frame rate of 40/sec for 3D speckle tracking strain analysis. The results of 18 myocardial segments were averaged to obtain global strain in longitudinal, circumferential, area, and radial directions. In addition to curves, numerical strain and timing data from each frame was obtained. Figure 1B demonstrates the diagram of the data acquisition set up for cardiac ultrasound examinations. The echocardiograph numerical data and the electro-mechanical signals were later manually synchronized during post-processing steps. We stored the corresponding ECG and MEMS data for each captured ultrasound image for all the subjects.

## Results

### Gyrocardiography Waveform Morphology

**Figure 2:**
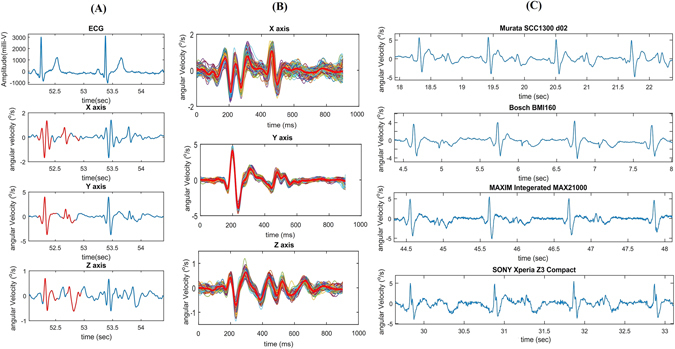
Typical three dimensional GCG waveforms from *x*, *y*, and *z* axes of rotation. 3-axis GCG morphologies and reference ECG (**A**). 3-axis ensemble averaged GCG morphologies (**B**). GCG *y*-axis waveforms obtained using different sensors (**C**).

Figure 2A represents reference ECG and corresponding three axis GCG angular velocity measurements caused by precordial vibrations. The GCG measures angular velocities with respect to three orthogonal axes of rotation, denoted by *x*, *y*, and *z*. Three dimensional GCG is achievable by a tri-axial gyroscope sensor. The axes of rotations are defined in this paper as follows: the *x*-axis points laterally from left to right, the *y*-axis points from head to foot, and the *z*-axis points from back to front. These axes of rotation are illustrated in the Fig. 1A.

As shown in the Fig. 2A and B, each GCG rotation axis corresponds to its own signal pattern (the most dominant patterns are highlighted by red color traces) with a magnitude in the order of few degree per second (dps or °/*s*). During systole, the angular velocity signal corresponding to the *x*-axis undergoes a fast and strong down-up-down deflection around the ECG R-wave, while a smaller upward deflection is seen around the T-wave. The *y*-axis consists of a repeating major peak that indicates the heartbeat pulse during systole. This prominent and upward spike is followed by again a minor upward deflection that appears slightly after the reference ECG T-wave. Similarly, the *z*-axis yields repeating waveforms for the systole and diastole; however, in this paper we will mainly focus on the waveforms corresponding to the *x*- and *y*-axes of the GCG, as these signals are typically of better quality.

The *x*- and *y*-axes of the GCG yield monomorphic patterns meaning that these waveforms are similar in shape with different subjects and measurement devices. As an example of this, Fig. 2C shows GCG signals measured from the *y*-axis of different sensors, namely Murata SCC1300d02, Bosch BMI 160, Maxim Integrated MAX21000, and the SONY Xperia Z3 compact. Although these sensors have diverse technical specifications in terms of noise level, power consumption, and full scale range, it can be seen that the obtained GCG signals are similar in amplitudes (in the scale of a few degree per seconds) and in the shapes of the waveforms; in particular the fiducial points described in the following section are visible in all of these signals.

### Gyrocardiography Waveform Annotation and Estimation of Hemodynamic Variables

Pulse wave Doppler images were obtained to define opening and closure times of the aortic and mitral valves from the considered 9 healthy subjects. We followed guidelines in ref. 42 to detect intra-cardiac events and correspondingly measure cardiac time intervals using mitral inflow and LV outflow velocity timings (See supplementary materials for more details).

**Figure 3:**
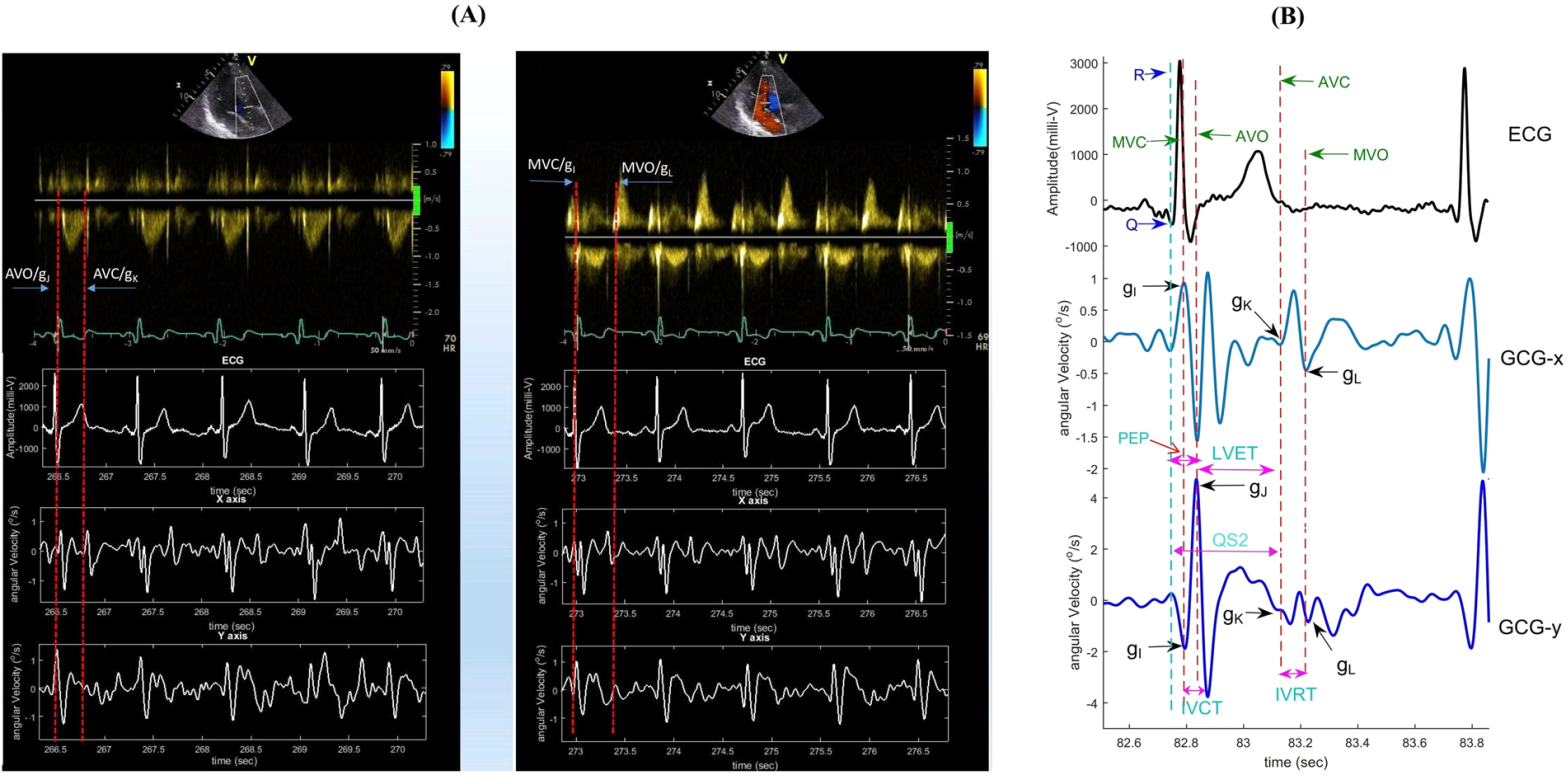
Waveform annotation and cardiac time interval estimation in GCG signal. Aortic (left) and mitral (right) valve opening and closure moments as measured by PW Doppler and correspondingly in GCG signal (**A**). Waveform annotation in GCG and corresponding time intervals with respect to ECG peaks (**B**).

Measurements of systolic and diastolic time intervals on the GCG signal requires robust delineation of cardiac fiducial points. Generally, the Q-peak and the R-peak in ECG serve as the reference points for measuring cardiac time intervals in echocardiography. Therefore, in this study we followed the same standard and measured time intervals from the ECG fiducial points to the considered GCG fiducial points. Our hypothesis for this research was that major stationary and repeating waveforms in GCG signal coincide with physiological events in heart. We performed visual inspections, through all study subjects and acquired data, in both PW Doppler images and GCG signals to identify specific GCG waveforms which coincide with particular mechanical cardiac events in each cardiac cycle (see Fig. 3A) and nominated each corresponding wave with a unique name as described below. Six major successive points, four of which coincide with valvular activity of the heart and two of which coincide with timings of maximal systolic and diastolic myocardial velocities, were identified as follows:

Around the ECG R-wave and during the systole, a fast downward notch in the *y*-axis wave pattern is visible. We denote this peak by *gyro*_*I*_ (*g*_*I*_). Right after *g*_*I*_, we denote the major maximum peak in the GCG *y*-axis signal by *gyro*_*J*_ (*g*_*J*_); this peak occurs slightly after the ECG R-wave. Further in the middle of the cardiac cycle and roughly after the ECG T-wave (around the second heart sound (S2)), a lower magnitude up-down wave (almost ∧ shape) is visible which consists of two reproducible and repeating notches just before and after the ∧ wave peak. This waveform is mostly visible in the signal obtained from the *x*-axis of the GCG, and we nominate the first notch by *gyro*_*K*_ (*g*_*K*_), and the second notch one by *gyro*_*L*_ (*g*_*L*_).

With distinguishing of GCG *g*_*I*_, *g*_*J*_, *g*_*K*_, and *g*_*L*_ points in every cardiac cycle, the isovolumetric contraction time (IVCT) and the isovolumetric relaxation time (IVRT) could be also estimated. Additionally, other significant systolic time intervals (STI) and indexes of cardiac contractility, including the total electromechanical systole (QS2), the left ventricular ejection time (LVET), and the pre-ejection period (PEP), could be estimated. In our considerations, the QS2 is measured from the ECG Q wave to the moment of *g*_*K*_ (AVO), while the LVET is measured as the time interval between the moments of AVO and AVC in the cardiac cycle, which in GCG corresponds to the time interval from *g*_*J*_ to *g*_*K*_. The PEP index can be determined by calculating the time between the ECG Q-wave and the onset of the aortic opening, which corresponds to *g*_*J*_ in GCG. PEP and LVET are both important clinical parameters on myocardial contractility^19, 43, 44^. Figure 3B illustrates the annotated GCG waveforms and the corresponding cardiac time intervals.

**Figure 4:**
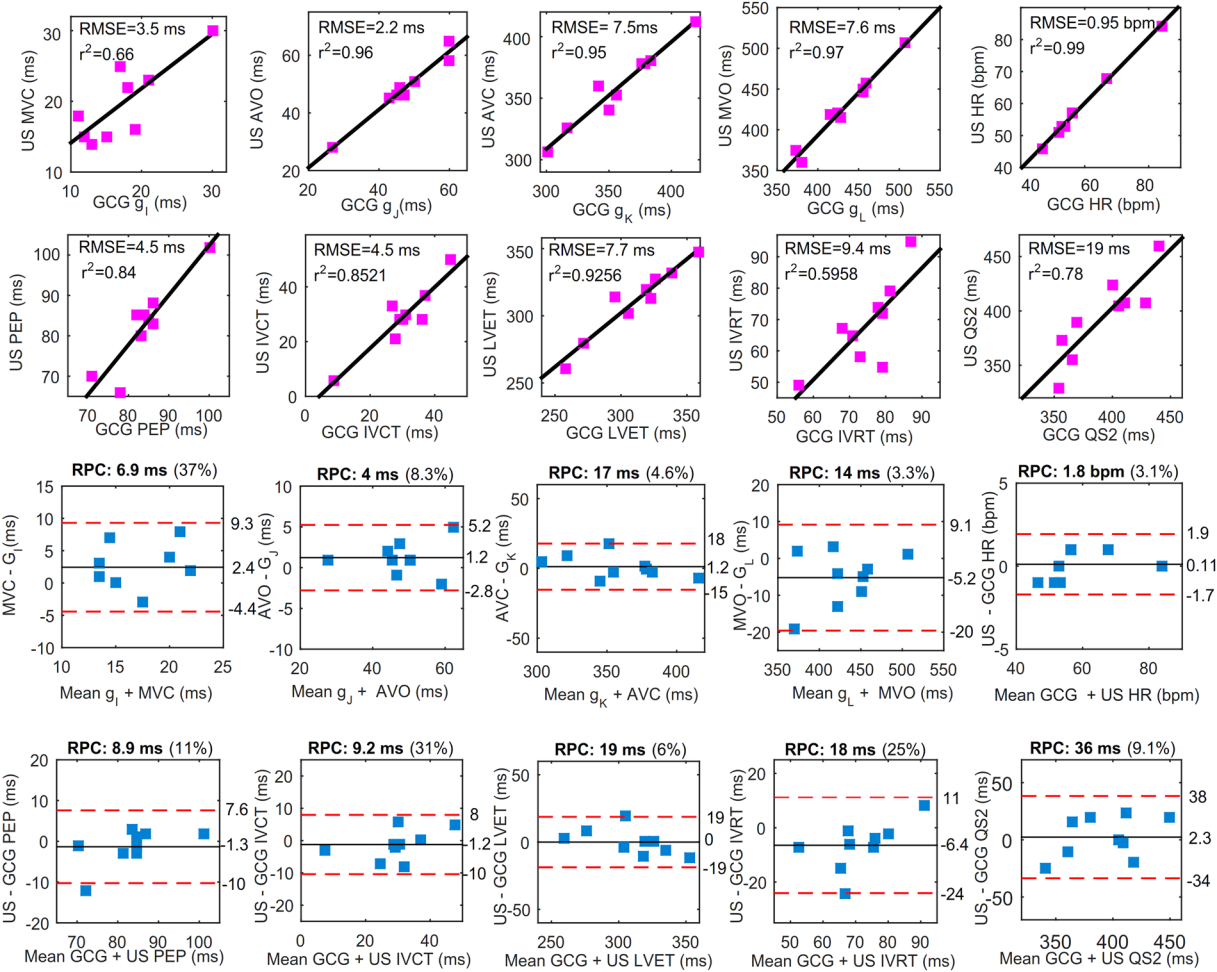
Correlation and Bland-Altman plots. The red color dashed lines drawn in Bland-Altman plots represent the upper and lower LoA ranges for the measured cardiac time intervals. RPC is the reproducibility coefficient value which is the maximum difference that is likely to occur between different observations. The coefficient of variation (CV) percentage is the ratio of the standard deviation and the overall mean.

We hypothesize that the fiducial points *g*_*I *_− *g*_*L*_ correspond to the opening and closing times of the heart valves. More specifically, *g*_*I*_ occurs approximately simultaneously with mitral valve closure (MVC), *g*_*J*_ with aortic opening (AO), *g*_*K*_ with aortic closure (AC), and *g*_*L*_ with mitral valve opening (MVO). In order to assess the validity of the hypothesis we compared the obtained GCG intervals to the reference tissue velocity signals measured by echocardiographic pulse wave interrogations. In all the following statistical analyses, we used Pearson correlation and Bland-Altman evaluation^45^ with 95% limits of agreement (LoA), corresponding to difference mean ±1.96× standard deviation. A Pearson correlation coefficient (*r* ^2^) was computed to assess the linear relationships. Positive correlation coefficients and root mean square error (RMSE) between the time intervals were obtained with all cardiac time intervals. Figure 4 shows linear association and agreement between the reference pulse wave- and measured GCG-based cardiac time intervals. The mean and SD of the measured GCG cardiac time intervals as well as their correlation and RMSE to the reference US measurements have been reported in Table 2.

**Table 2:** US and GCG derived information for underlying cardiac time intervals.

US	Mean ± SD	GCG	Mean ± SD	r^2^	RMSE
HR (bpm)	59 ± 12	HR	58 ± 11	0.99	0.95
R-MVC (ms)	20 ± 6	R-*g*_*I*_	18 ± 5	0.66	3.5
R-AVO (ms)	49 ± 10	R-*g*_*J*_	47 ± 10	0.96	2.2
R-AVC (ms)	359 ± 32	R-*g*_*K*_	358 ± 36	0.95	7.5
R-MVO (ms)	427 ± 44	R-*g*_*L*_	432 ± 41	0.97	7.6
IVCT (ms)	29 ± 12	IVCT	30 ± 10	0.85	4.5
IVRT (ms)	68 ± 14	IVRT	74 ± 9	0.59	9.4
QS2 (ms)	393 ± 34	QS2	394 ± 38	0.78	19
LVET (ms)	310 ± 26	LVET	310 ± 32	0.93	7.7
PEP (ms)	82 ± 10	PEP	83 ± 8	0.84	4.5
Q-Sa (ms)	134 ± 20	Q-SPV	132 ± 26	0.89	7.6
Q-Ea (ms)	467 ± 44	Q-DPV	460 ± 49	0.87	16
Q-Max Strain (ms)	367 ± 32	Q-Max Ang Disp	371 ± 31	0.91	10

### Estimating timings of peak myocardial movements and deformations

#### Tissue velocity and Strain echocardiography

In echocardiography myocardial motions and deformations can be measured for example by tissue velocity and strain measurements. For this part of the work, we obtained echocardiographic images from 9 healthy subjects, and measured myocardial longitudinal wall motion and deformation using tissue Doppler imaging (TDI) and three dimensional (3D) speckle tracking, respectively. In TDI images, multiple regions of interest (ROI) were placed in the left ventricle myocardium in apical 4-chamber view in order to measure average myocardial velocity and displacement. As the gyroscope signal is a velocity signal, it is natural to look at the correspondence between the GCG signal and the tissue velocity acquired by the echocardiograph. In this work we consider only correspondence in time, that is, how the timings of the peaks in GCG signal are correlated with the timings of the peak tissue velocities. However, an interesting future research topic is to investigate how the waveforms themselves are correlated and what information can be gained from the magnitude of the GCG signal. Currently, it is not known how the GCG signal attenuates due to the tissue between the heart and the sensor. Accurate estimation of the timing of the maximal tissue velocities, can, however be clinically important, as it enables for example the computation of the myocardial dispersion which is the standard deviation of time to maximum myocardial shortening. Myocardial dispersion reflects the heterogeneity of myocardial systolic contraction and can be used as an indicator for susceptibility to arrhythmias in different heart disease groups such as heart failure, ischemia, and infarction^46^.

**Figure 5:**
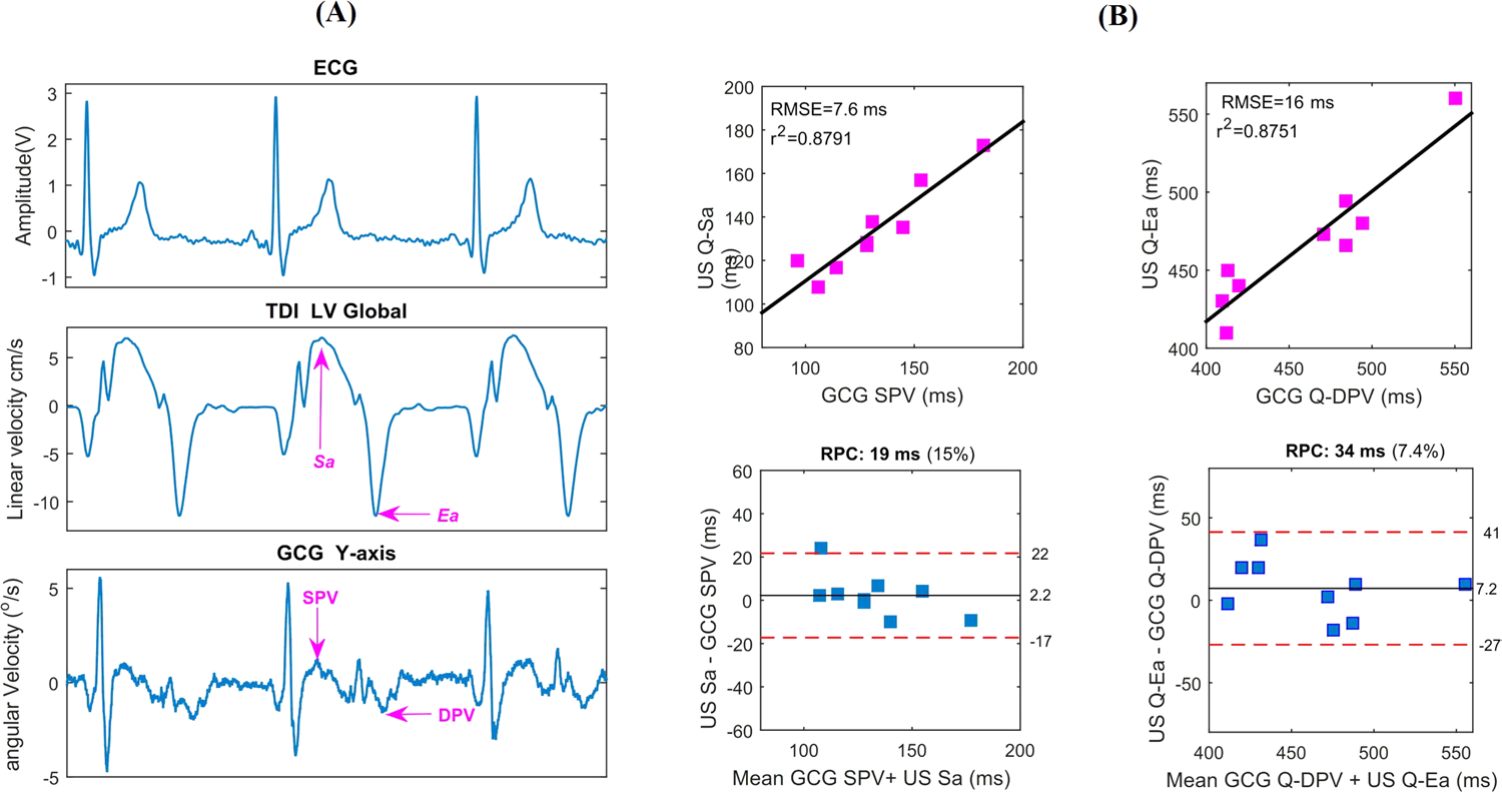
*Sa* and *Ea* wave evaluations with TDI and GCG. Qualitative comparison between the TDI *Sa* and *Ea* waves and corresponding *SPV* and *DPV* in typical GCG y- and z-axis waves (**A**). Quantitative evaluation of time intervals between Q-*Sa*/*Ea* versus Q-*SPV*/*DPV* waves (**B**).

We noticed a relatively wide positive polarity waveform (as shown in Fig. 5A) which appears in the GCG *y*-axis signal after the R-peak in ECG and the fiducial point *g*_*J*_. Another repeating waveform in the *y*-axis signal is a minor V-shaped dip-rise wave which appears soon after *g*_*L*_. As shown in Fig. 2C, also these waveforms are reproducible using different types of gyroscopes. We called these two repeating waveforms the *systolic peak velocity* (SPV) and the *diastolic peak velocity* (DPV), respectively. As is visible from Fig. 5A, the SPV point occurs approximately at the time of systolic myocardial velocity (*Sa*) point in TDI, while the timing of DPV coincides approximately with the early diastolic velocity (*e*′ or *Ea*) in TDI.

The time intervals from ECG-Q wave to GCG *SPV*/*DPV* waves and ECG-Q to *Sa*/*Ea* waves were measured for the considered 9 healthy subjects (see Table 2). Figure 5B shows statistical analysis for the these time intervals. Linear correlation of *r* ^2^ = 0.88 and agreement of (upper and lower LoA^22, 45^: ms and [−17, −27] ms, respectively) were achieved. Clinically *Sa* and *Ea* are important, as *Sa* is a measure of longitudinal systolic function and is correlated with EF and peak *dP/dt*, while *Ea* is a marker of diastolic function.

**Figure 6:**
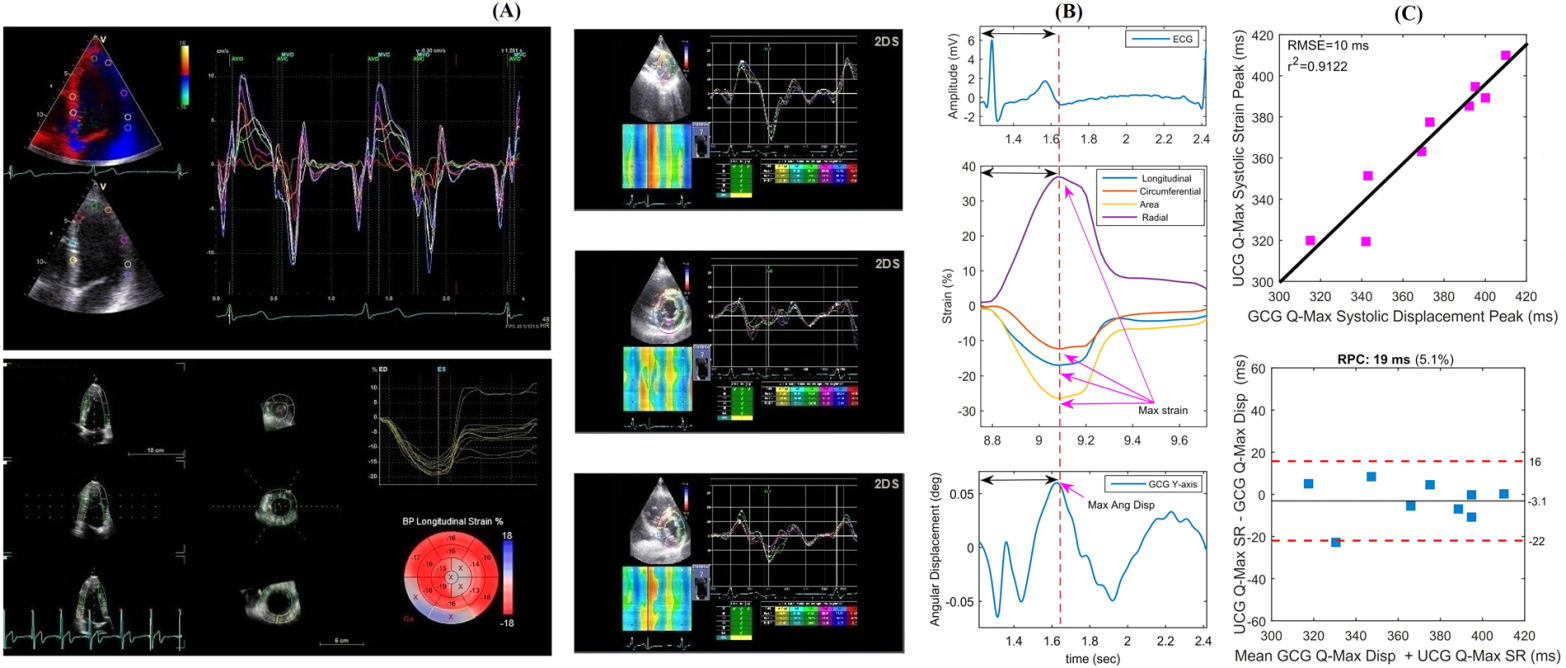
Myocardium tissue velocity, displacement, and strain analysis using TDI and corresponding GCG based angular rates (y-axis). Tissue velocity and displacement using TDI and 3D speckle tracking strain in longitudinal, circumferential and radial directions (**A**). Electromechanical delays measured by TDI and GCG (**B**). Relationship between the GCG and TDI electromechanical delays (**C**).

In addition to the tissue velocity, we also assessed the myocardium deformation by measuring global speckle tracking 3D strain, which is the fractional change in length of the myocardium either in radial, longitudinal or circumferential dimension. Strain is a function of position, that is, the integral of velocity, and therefore we compared the strain measurements to the integral of the GCG *y*-axis signal, which we call here *angular displacement*. Figure 6A shows tissue velocity, that is longitudinal rate of tissue changes (upper sub-part), and corresponding myocardial strain curve (leftmost bottom and right side sub-parts), obtained by speckle tracking. Figure 6B shows the electromechanical delay from ECG Q-wave to the maximum global longitudinal, circumferential, area, and radial strains (middle sub-part) and in GCG from Q to the maximal angular displacement (bottom sub-part). The double arrows in this figure show that the time from ECG onset Q to the maximal strain approximately coincides with the time from ECG onset Q to maximal GCG angular displacement in *y*-axis. The average Q-maximal strain and Q-maximal angular displacement were 367 ± 32.2 ms and 371 ± 31.9, respectively. Moreover, Fig. 6C shows linear correlation and agreement between these timings obtained with 3D speckle tracking strain and GCG displacement curves, showing that maximal angular displacement points may be useful for estimating the myocardial mechanical dispersion. This is a possible application for GCG, as beat-by-beat evaluation of the electromechanical delay with GCG may bring new insights into the assessment of myocardial function. Measurements of mechanical dispersion can yield significant information about the risk of arrhythmia specifically with post myocardial infarction patients^46^.

### Visual Comparison of GCG and SCG

Thoracic vibrations in three-dimensional space consists of translation and rotation in three orthogonal directions. Translational quantities such as linear velocity and acceleration describe linear motions and are measured by for example an accelerometer sensor, while, as proposed in this study, rotational quantities such as angular displacement, angular velocity, and angular acceleration can be measured by a gyroscope sensor. Chest-accelerometry, also known as seismocardiography, is determined to generate a signal that is indicative of linear thoracic vibrations in response to heart’s contraction and the ejection of blood from the ventricles into the vascular tree^15^, whereas chest gyrocardiography that comprises a sensor of angular motion indicates rotational precordial movement or vibration on the chest in response to myocardium movements.

**Figure 7:**
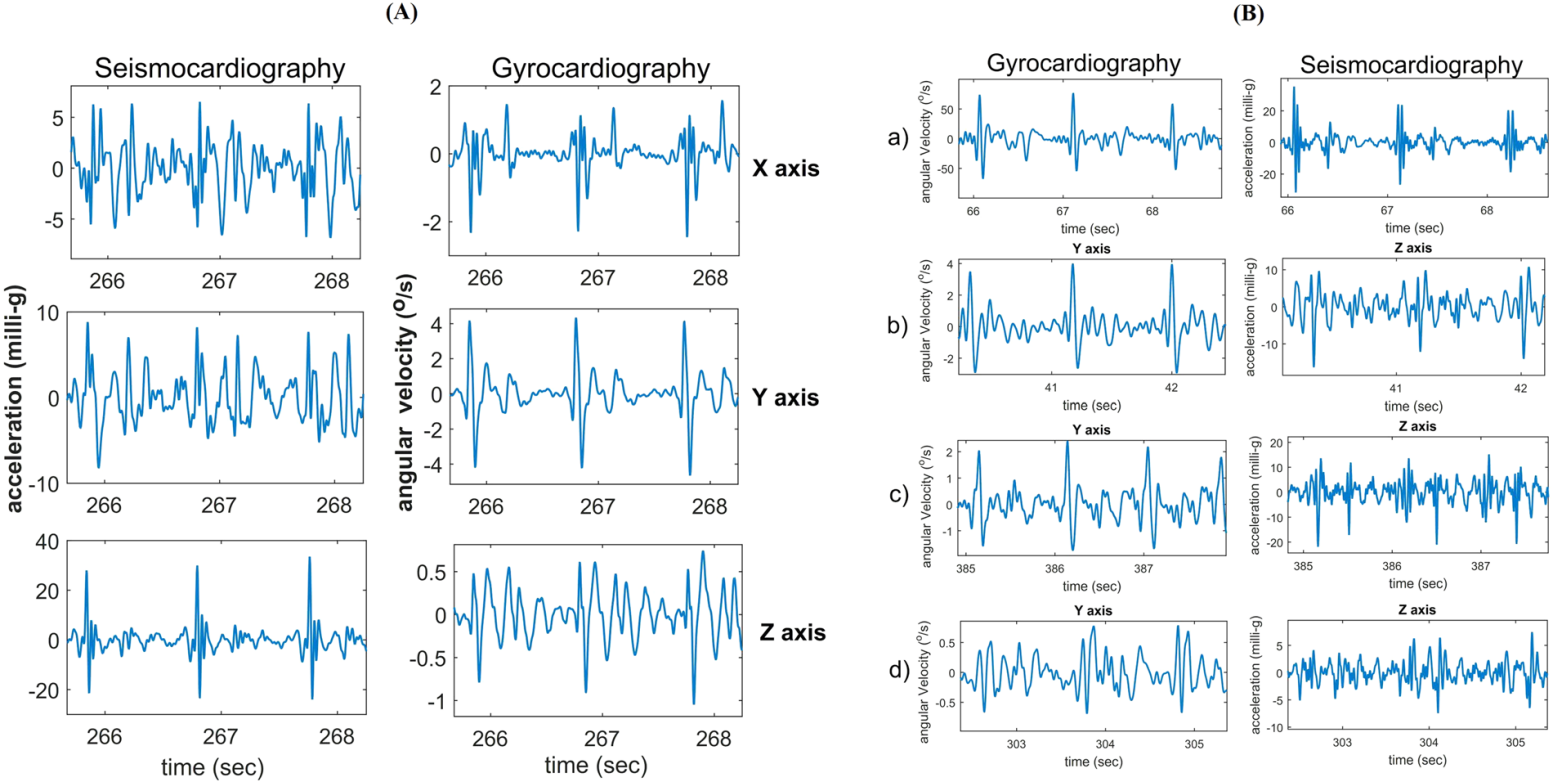
Visual comparison of GCG and SCG signals. Evaluation of signal quality in typical tri-axial SCG and GCG waveforms (**A**). Intersubject variability comparison for GCG against SCG (**B**).

We mainly considered waveforms characterized by several peaks and valleys, reflecting certain cardiophysiological events of the beating heart, on SCG amplitude of the dorso “ventral component (z-axis) and GCG amplitude of head-to-foot component (y-axis) in order to evaluate linear acceleration and rotational velocity vector trajectories during the heart cycle. Nevertheless, it is likely that other physiological information could be extracted also from the analysis of other GCG-SCG components. Our experience with GCG^39, 40, 47, 48^, has shown that gyrocardiography is less sensitive than seismocardiography to intra-subject and inter-subject variability in the morphology in cardiac signals. Figure 7A represents an example of 6-axis motion sensing using a 3-axis accelerometer and a 3-axis gyroscope. As shown, accelerometer-based measurements in all axes contain more noise as compared to the corresponding filtered GCG signals. Further, visual evaluation of waveforms in Fig. 7B implies that GCG is notably tolerant to inter-subject variability as sorted SCG-GCG signals — in terms of signal quality (top-down: good, medium, low, and very low) in four different subjects — indicate that GCG stays stationary and uniform (see insets a–d) while it is hardly possible to distinguish underlying waveforms in the very low quality SCG (e.g. see the inset d in Fig. 7B). Robustness against intra- and interpersonal variation is an advantage of GCG which makes it potentially useful for wearable cardiac monitoring.

## Discussion

Our major contributions in this work are the waveform annotation of a cardiac signal measured unobtrusively using a gyroscope, and the use of such signal for the estimation of the timing of maximal tissue velocity and strain of the myocardium measured with echocardiography. Our investigations show that the proposed method, GCG, can provide useful information related to the mechanical performance of the heart.

Automatic segmentation and delineation of the GCG signals depends on the reliable detection of heartbeats. We developed an automated heart beat detection based on Hilbert transform and provided a primary waveform annotation algorithm for beat-to-beat assessment of cardiac time intervals and estimation of STI related hemodynamic variables in GCG. For further details on the implementation and analysis of the segmentation and waveform annotation, readers can refer to the supplementary material. GCG allows continuous or frequent heart monitoring for the estimation of hemodynamic variables and can be used for heart arrhythmia detection. For example, in ref. 48 we have presented a method for automated detection of atrial fibrillation that is based on the estimation of the variation of the timings and amplitudes of GCG heartbeats. On the other hand, accurate and reliable PEP estimation is important since it allows to assess myocardial contractility affected by the cardiac preload and afterload. This index is relatively independent from the vagal drive and the heart rate. In patients with left ventricular failure, PEP increases because of the low contractility caused by the myocardial dysfunction^19, 43, 44^. LVET is also an important index of contractility, which unlike PEP, is influenced by the heart rate^43^.

Clinical value of SCG- and GCG-based tissue velocity measurement using multi-dimensional motion sensing has been previously addressed in refs 31, 48 and 49. For example, it has been shown that using machine learning and pattern recognition techniques irregular heartbeat (arrhythmia) as well as abnormality in mechanical performance of the myocardium (as a result of ischemic diseases) can be recognized^48, 49^. Namely, 6-axis motion sensing using joint multi-axial accelerometer and gyroscope sensors, based on incorporated IMUs either in smart devices sensors or in customized biomedical monitoring devices, can yield significant mechanical information – in time and frequency domains – of the heart function not obtainable by ECG alone.

Due to the potential advantages of personalized health monitoring systems, a growing number of mobile/wearable devices would benefit from reliable monitoring of the heart. A personal smart monitoring platform can assess the health risks by early detection of the cardiovascular disorders. Recent advances in the development of electromechanical sensors have resurged mechanocardiography techniques for clinical and non-clinical considerations. For instance, MEMS gyroscope and accelerometer can be either embedded into a monitoring patch device for long term usage^50–52^, or be employed from smart devices. These sensors are not subjected to intervention from electrical monitoring or implantable stimulating signals generated by ECG, pacemakers, and cardioverter defibrillators and therefore may be used for wearable continuous cardiac function monitoring in the future^53–55^.

The main limitation of this study is that only nine healthy subjects were examined; this effects the statistical power of our outcomes. However, the results are promising, and warrant subsequent measurements and analysis. A smaller problem with the experimental setup was that the movements of the ultrasound probe generated artefacts in the GCG signal. Moreover, GCG and echocardiography signals were synchronized using an external clock, which yields some small random delay between the signals. The TDI and 3D speckle tracking measurements were performed with an average frame rate of 106 ± 21 fps (frames per second) and 40 ± 10 fps, meaning that each frame contains information over 9.5 ms and 25 ms, respectively. It should be noted that in most of the cases considered in this work, the calculated RMSE values fall within the duration of a single frame.

A research direction for future is to develop advanced algorithms for automatic annotation of the wearable GCG signal using signal processing and machine learning approaches. Also, the potential of GCG for automated cardiac disease diagnostics will be considered. Sensor fusion algorithms using both SCG and GCG signals, and their clinical applications, will also be investigated in future studies. We should point out that although the measurements were performed concurrently using a 3-axis accelerometer and a 3-axis gyroscope, in this paper we focus on the novel properties of the gyrocardiograph. Nevertheless, we briefly performed primary comparisons on differences and similarities between SCG and GCG.

In conclusion, in this paper we have presented a new cardiac monitoring technique called gyrocardiography which is based upon a tri-axial gyroscope sensor and measures angular velocities of the chest as a response to the rotation of the heart. As shown in this paper, a gyroscope can accurately detect very small angular displacements with high temporal resolution, and thereby it is capable of revealing precordial micro-vibrations caused by myocardial motions. Our observations indicated that the morphology of the GCG signal is reproducible with different gyroscopes. Accordingly, we explored the feasibility of GCG waveform annotation on underlying systolic and diastolic repeating patterns and indicated that tri-axial GCG provides reliable fiducial points for cardiac events. Complementary statistical evaluations then revealed that the GCG signal is able to give reliable information on cardiac time interval measurements such as systolic time intervals (STI) and diastolic time intervals (DTI). STIs including left ventricular ejection time and pre-ejection period can be measured by detecting particular indicative mechanical cardiac events, for example, instants of MC, AO, MO, and AC in GCG signal. Moreover, newly-identified GCG points, i.e. *SPV* and *DPV*, are indicative of systolic myocardial velocity and early diastolic velocity (as research showed good temporal correlations between GCG and US velocity measurements) and can potentially provide functional information related to systolic and diastolic activities. We also indicated that the time from ECG onset Q to the maximal TDI strain is correlated to the time interval from ECG onset Q to maximal point of GCG angular displacement. This electromechanical delay may bring new insights into the assessment of myocardial function as its variation, known as myocardial mechanical dispersion, can potentially help in detection of arrhythmias and myocardial infarction^46^. Therefore, wearable/mobile GCG as a promising mechanical cardiac monitoring tool can be used in quantification of beat-by-beat dynamics of cardiac time intervals and can potentially represent information related to the hemodynamic variables and myocardial contractility.

## Electronic supplementary material

Supplementary information
