# Supplementary material for: Gyrocardiography: A New Non-invasive Monitoring Method for the Assessment of Cardiac Mechanics and the Estimation of Hemodynamic Variables
Source: Sci Rep. 2017 Jul 28;7:6823. doi: 10.1038/s41598-017-07248-y (PMC5533710; doi:10.1038/s41598-017-07248-y)
Supplement: Supplementary file 1 — Supplementary information [file 41598_2017_7248_MOESM1_ESM.pdf]

Gyrocardiography: A New Non-invasive Monitoring Method for the Assessment of Cardiac Mechanics and the Estimation of Hemodynamic Variables

Mojtaba Jafari Tadi[1,2*], Eero Lehtonen[2], Antti Saraste[1,3], Jarno Tuominen[2], Juho Koskinen[2], Mika Teräs[4,5], Juhani Airaksinen[1,3], Mikko Pänkäälä[2], and Tero Koivisto[2]

[1]University of Turku, Department of Cardiology and Cardiovascular Medicine, Turku, Finland
[2]University of Turku, Department of Future Technologies, Turku, Finland
[3]Turku University Hospital, Heart Center, Turku, Finland
[4]University of Turku, Institute of Biomedicine, Turku, Finland
[5]Turku University Hospital, Department of Medical physics, Turku, Finland
[*]mojtaba.jafaritadi@utu.fi

ABSTRACT

In this supplementary material, additional experimental results on gyrocardiography (GCG) are presented. An automated heartbeat detection and cardiac cycle extraction algorithm for the GCG is presented. Furthermore, a method for automated determination of cardiac time intervals using GCG is described. The presented methods can be used for example in continuous wearable monitoring applications of GCG.

Supplementary Materials

Automated heartbeat detection using GCG

A group consisting of 29 healthy subjects was used for concurrent GCG and ECG measurements for automated heartbeat detection and cardiac cycle extraction. ECG was used here only for reference and validation; the presented automated heartbeat detection method operates solely on the GCG signal, and does not need ECG for extracting fiducial points. Demographic information of test subjects has been provided in Table 1.

Based on our previously published method for SCG[1], we developed an algorithm that automatically detects g_J points from the y-axis signal of the GCG. Our method is based on applying adaptive thresholds on GCG signal that has been processed using the Hilbert transform; this Hilbert adaptive beat identification technique (HABIT) is described in detail in[1]. For comparison, we measured simultaneously ECG Lead II from the test subjects, and applied the Pan-Tompkins[2] method for extracting the heart beats from the ECG signal. The performance of our GCG heart beat estimation algorithm was tested against ECG in three different positions, i.e. left recumbent, right recumbent, and supine. Concurrently with the SCG measurements presented in[1], we also performed GCG measurements with MAX21000 gyroscope, and the heartbeat detection algorithm was applied to this data. All the data acquisitions were made up to 5 minutes for each subject per position. Fig. 1a shows heartbeat detection using our method. Fig. 1b shows the correlation ($r^2 > 0.99$) and agreement between GCG and ECG interbeat intervals for all measured positions (left n=6, right n=6, and supine n=29) — where n is the number of test subjects. Potential applications for beat-by-beat estimation using GCG include heart rhythm assessment, heart rate variability (HRV) analysis[3], and medical imaging[4].

The performance of the cardiac cycle estimation using HABIT was assessed and computed separately for each subject using different statistical parameters such as Pearson's correlation, Bland-Altman plot, and root mean square error (RMSE). Additionally, we calculated the heartbeat detection ability of the method by evaluating precision or positive predictive value

Table 1. Demographic information of the study subjects as reported in[1]

Demographic information (n=29)	Min	Max	Average	SD
Age (years)	23	41	29	4
Height (cm)	170	190	178	5
Weight (kg)	60	98	76	11
BMI (kg/m^2)	17.5	29.4	23.9	3

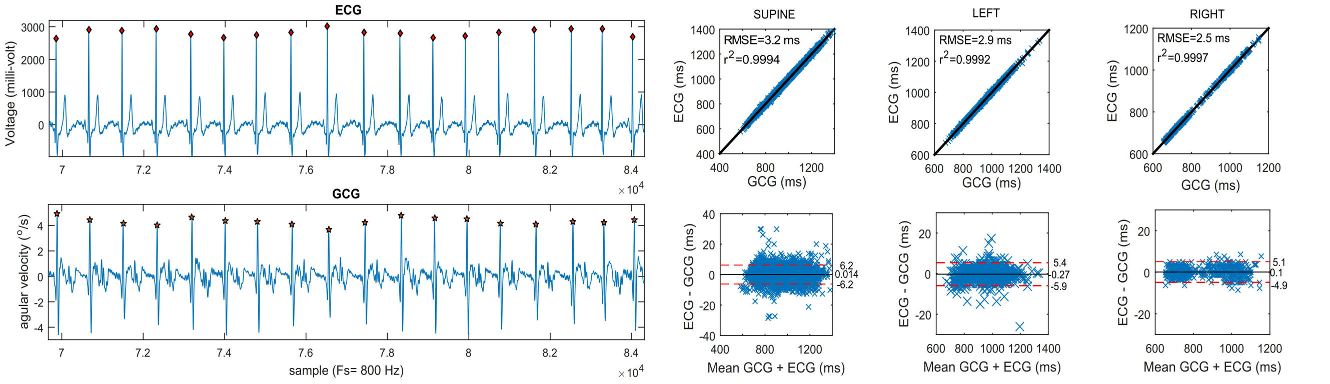

(a) Adaptive heartbeat detection in GCG $y-axis$.

(b) Correlation and agreement between the ECG and GCG in three different positions.

Figure 1. Heartbeat detection using GCG and reference ECG.

(PPV) and sensitivity or true positive rate (TPR). Sensitivity and precision evaluate the capability of the detector to find valid heart beats and to reject incorrectly detected beats as follows:

$$\text{Sensitivity or TPR }(\%) = \frac{\text{TP}}{\text{TP}+\text{FN}} \times 100, \tag{1}$$

$$\text{Precision or PPV }(\%) = \frac{\text{TP}}{\text{TP}+\text{FP}} \times 100, \tag{2}$$

where TP represents the total number of true positives, FN the total number of false negatives, and FP the total number of false positives. Detected heart peaks within a window of ±150 ms around each R peak were considered true positive. Conversely, if no valid R peak was found to be associated to the detected pulse, the unassigned GCG pulse peak was considered false positive, while for any valid R peak to which no pulse peak was assigned, one false negative is counted.

The average true positive rate (TPR), positive prediction rate (PPR) and detection error rate (DER) for different positions were as follows (TPR, PPR, and DER): supine (99.4%, 99.5% and $\simeq$ 0.038%), left (99.66%, 99.7% and $\simeq$ 0.034%) and right (98.9%, 96.6%, and $\simeq$ 0.049%). The average beat-to-beat interval and root mean square error (RMSE) between ECG (R-R) and GCG (g_J-g_J) interbeats were as follows: 961$\pm$ 12 ms and 3.2 ms in supine, 938$\pm$ 117 ms and 2.9 ms in right recumbent, and 963$\pm$99 ms and 2.5 ms in left recumbent positions, respectively.

Time Interval Measurements in Doppler Pulse Wave and GCG

Cardiac time intervals were measured using mitral inflow and LV outflow velocity timings using Doppler pulse wave technique and according to[5] guidelines. The mitral inflow velocity waves were obtained with a sample volume positioned between the tips of the mitral leaflets. The LV outflow patterns were achieved by positioning sample volume (cursor) below the aortic valve. Fig. 2a shows the situation of mitral inflow and LV outflow. The interval 'a' was measured from the termination to the onset of mitral inflow, while the interval 'b', the duration of left ventricular velocity profile or LVET, was measured from the onset to the cessation of LV outflow. The interval 'c' was measured from the ECG R-peak (reference point) to the cessation of the LV outflow or AVC, while the interval 'd' was measured from the R-peak to the onset of the mitral inflow (MVO). The interval 'e' was measured from the ECG Q point to the end of the LV outflow, representing QS2 distance. IVRT was measured by subtracting 'd' from 'c'. PEP was calculated by subtracting 'b' from 'e' and IVCT by subtracting IVRT and 'b' from 'a'.

Automated Waveform Annotation and CTI Estimation in GCG

As described in the main manuscript, by identifying from the GCG signal the fiducial points g_I, g_J, g_K, and g_L, we can estimate systolic and diastolic time intervals such as IVCT, QS2, LVET, PEP, and IVRT. We developed an automated method – based on the algorithms described in[6,7] – for identifying the four fiducial GCG points g_I-g_L from the x- and y-axis signals.

The g_J peak in GCG y-axis and diastolic $\wedge$-wave peak (nominated as the local maxima in early diastolic phase visible in GCG x-axis) serve as the reference points in every GCG cardiac cycle. Therefore, the first task is to detect these points as described above for the automated heartbeat detection – as labeled by diamond signs in Fig. 2b) – from the GCG x- and y-axis. Then, two search windows W1 and W2 are defined with the help of g_J peaks. W1 starts from the onset of g_J-peak in GCG

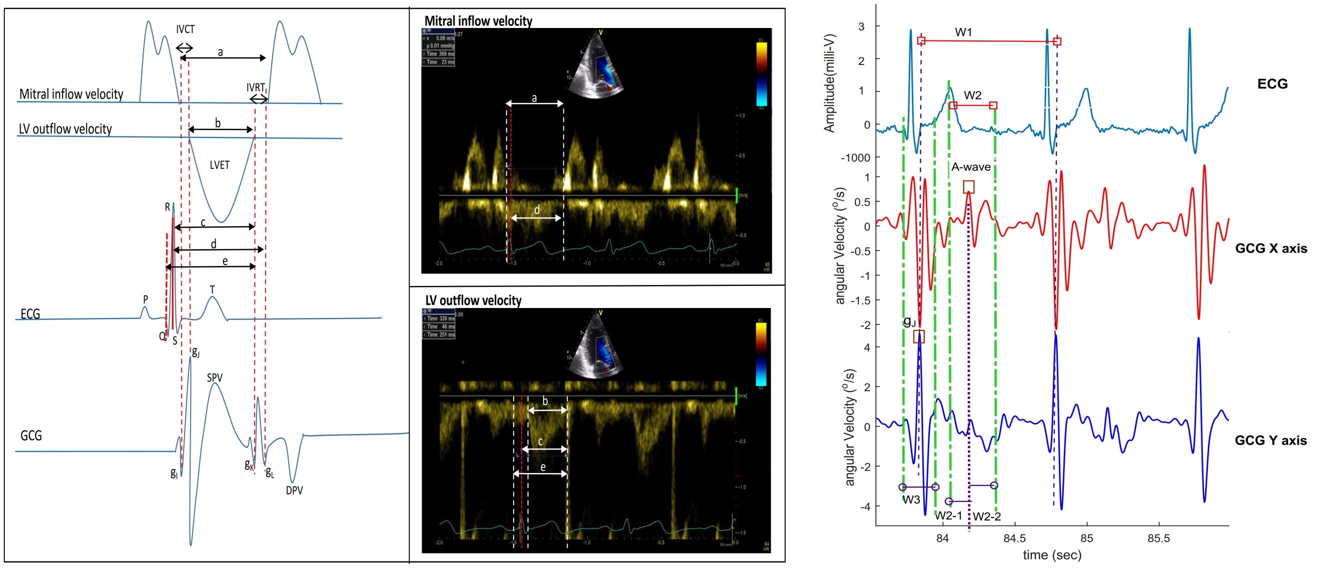

(a) Doppler and GCG time intervals measurements.

(b) GCG waveform delineation according to PW Doppler and observational interpretation.

Figure 2. Estimation of cardiac events in reference Doppler images and in GCG x- and y-axis

y-axis to the onset of the following g_J. Within this window, we define W2 (a 200 ms long search window starting from 250 ms after the previously detected g_J) and search for the local maxima, also called $\wedge$-wave, in the GCG x-axis. Subsequently, we define sub-windows W2-1 and W2-2 (each of length 100 ms) to search for the neighboring minima located in the right and left side of the A-wave. The local minimum points identified within W2-1 and W2-2 refer to g_k and g_L, respectively. Similarly, we define a 200 ms long search window W3 backwards from g_J to the left (-100 ms) to find local minimum neighbour, known as g_I, and forwards to the right (+100 ms) to localize Max-velocity point. Likewise, we identify fiducial points using a primary windowing technique and afterward estimate the cardiac time intervals. Fig. 3 shows automated waveform annotation in GCG x- and y-axis using the explained method.

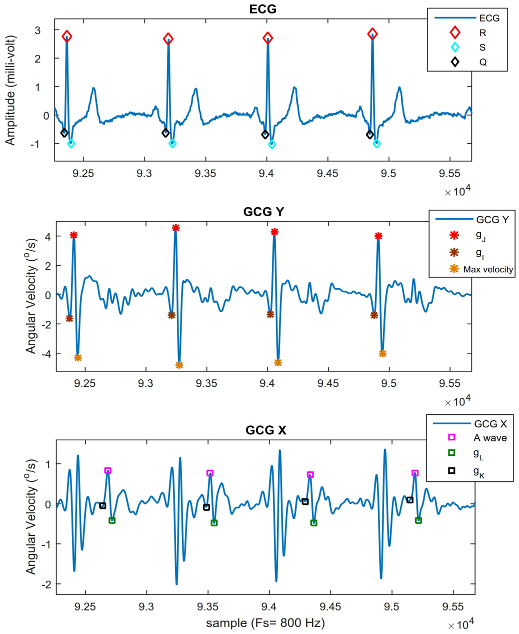

Figure 3. Automated heartbeat detection using HABIT and correspondingly delineated cardiac events in GCG x- and y-axis

References

1. Jafari Tadi, M. *et al.* A real-time approach for heart rate monitoring using a Hilbert transform in seismocardiograms. *Physiological Measurement* **37**, 1885 (2016).
2. Pan, J. & Tompkins, W. J. A Real-Time QRS Detection Algorithm. *Biomedical Engineering, IEEE Transactions on* **BME-32**, 230–236 (1985).
3. Lahdenoja, O., Hurnanen, T., Tadi, M. J., Pänkäälä, M., Mikko & Koivisto, T. Heart rate variability estimation with joint accelerometer and gyroscope sensing. In *Computing in Cardiology, 2016*, vol. 43, 1–4 (2016). URL http://www.cinc.org/archives/2016/pdf/209-166.pdf.
4. Jafari Tadi, M. *et al.* A miniaturized MEMS motion processing system for nuclear medicine imaging applications. In *Computing in Cardiology, 2016*, vol. 43, 1–4 (2016). URL http://www.cinc.org/archives/2016/pdf/042-452.pdf.
5. Tekten, T., Onbasili, A. O., Ceyhan, C., Ünal, S. & Discigil, B. Novel approach to measure myocardial performance index: Pulsed-wave tissue doppler echocardiography. *Echocardiography* **20**, 503–510 (2003).
6. Khosrow-khavar, F. *et al.* Automatic annotation of seismocardiogram with high-frequency precordial accelerations. *Biomedical and Health Informatics, IEEE Journal of* **19**, 1428–1434 (2015).
7. Jafari Tadi, M. *et al.* A new algorithm for segmentation of cardiac quiescent phases and cardiac time intervals using seismocardiography. In *Proc. SPIE, Sixth International Conference on Graphic and Image Processing (ICGIP)*, vol. 9443, 94432K–94432K–7 (2015).
